# Predictive Significance of Kidney Myeloid-Related Protein 8 Expression in Patients with Obesity- or Type 2 Diabetes-Associated Kidney Diseases

**DOI:** 10.1371/journal.pone.0088942

**Published:** 2014-02-18

**Authors:** Takashige Kuwabara, Kiyoshi Mori, Masato Kasahara, Hideki Yokoi, Hirotaka Imamaki, Akira Ishii, Kenichi Koga, Akira Sugawara, Shinji Yasuno, Kenji Ueshima, Takashi Morikawa, Yoshio Konishi, Masahito Imanishi, Akira Nishiyama, Kazuwa Nakao, Masashi Mukoyama

**Affiliations:** 1 Department of Medicine and Clinical Science, Kyoto University Graduate School of Medicine, Kyoto, Japan; 2 Medical Innovation Center, Kyoto University Graduate School of Medicine, Kyoto, Japan; 3 Department of EBM Research, Institute for Advancement of Clinical and Translational Science, Kyoto University Hospital, Kyoto, Japan; 4 Department of Nephrology, Osaka Red Cross Hospital, Osaka, Japan; 5 Division of Nephrology and Hypertension, Osaka City General Hospital, Osaka, Japan; 6 Department of Pharmacology, Kagawa University Medical School, Kagawa, Japan; University of Louisville, United States of America

## Abstract

**Background and Objective:**

We have reported that toll-like receptor 4 (TLR4) and one of its endogenous ligands, myeloid-related protein 8 (MRP8 or S100A8), play an important role in the progression of diabetic nephropathy in mice. The aim of this study was to evaluate significance of kidney MRP8 expression in patients with obesity- or type 2 diabetes-associated kidney diseases.

**Methods:**

In diabetic, obese or control subjects, MRP8 mRNA and protein expression levels in renal biopsy samples were determined by real-time RT-PCR and immunohistochemistry (n = 28 and 65, respectively), and their associations with baseline and prognostic parameters were analyzed. Effects of MRP8 upon pro-inflammatory gene expressions were examined using macrophages.

**Results:**

Kidney MRP8 gene and protein expression levels were elevated in obese or diabetic groups compared to control group. Among all subjects, by univariate linear regression analysis, glomerular MRP8-positive cell count and tubulointerstitial MRP8-positive area at baseline were both, respectively, correlated not only with various known risk factors for diabetic nephropathy (such as systolic blood pressure, proteinuria and serum creatinine) but also with extent of glomerulosclerosis and tubulointerstitial fibrosis. Independent factors predicting urinary protein levels a year later were examined by multivariate analysis, and they included glomerular MRP8-positive cell count (β = 0.59, P<0.001), proteinuria (β = 0.37, P = 0.002) and systolic blood pressure (β = 0.21, P = 0.04) at baseline, after adjustment for known risk factors. MRP8 protein expression was observed in CD68-positive macrophages and atrophic tubules. In cultured mouse macrophages, MRP8 protein induced proinflammatory cytokine expressions and also triggered auto-induction of MRP8 in a TLR4-dependent manner.

**Conclusions:**

Glomerular MRP8 expression appears to be associated with progression of proteinuria in obese or type 2 diabetic patients, possibly by inducing inflammatory changes in macrophages through TLR4 signaling.

## Introduction

Chronic inflammation plays an important role in the pathogenesis of diabetes or obesity and their cardiovascular complications [1]. Involvement of innate immune receptors and the endogenous ligands in the process of chronic inflammation has been implicated. Myeloid-related protein 8 (MRP8, also known as S100A8 or calgranulin A) was originally identified as a cytoplasmic calcium-binding protein in neutrophils and monocytes [2], and has become widely recognized as a potent endogenous ligand for toll-like receptor 4 (TLR4) in various diseases including septic shock, vascular and autoimmune disorders [3], [4], [5]. We have recently proposed that MRP8/TLR4 signaling plays an important role in hyperlipidemia-induced progression of diabetic nephropathy [6]. Glomerular macrophages and collecting duct cells are major sources of MRP8 in mouse models of diabetic nephropathy [6] and renal fibrosis [7], respectively. Plasma levels of MRP8, which usually forms a heterodimeric complex with a binding partner MRP14 in the bloodstream, are increased in obese subjects [8], [9]. However, there have been no reports investigating renal expression of MRP8 in patients with obesity or type 2 diabetes and its association with renal prognosis.

The aim of this study was to determine mRNA and protein expression levels of MRP8 in the kidney of Japanese patients with diabetic nephropathy (DN), obesity-related glomerulopathy (ORG), minimal change nephrotic syndrome (MCNS) or minor glomerular abnormality (MGA), which were all diagnosed by renal biopsy, and to evaluate whether renal MRP8 expression can predict renal outcomes.

## Materials and Methods

### Ethics statement

The human study was conducted according to the principles expressed in the Declaration of Helsinki, and was approved by the Ethical Committees on Human Research of Kyoto University Graduate School of Medicine and Osaka City General Hospital, respectively. All participants gave written informed consent. The animal study protocol was approved by the Animal Research Committee of Kyoto University Graduate School of Medicine (Permit Number: Med Kyo 13318). All animal surgery was performed under sodium pentobarbital anesthesia, and all efforts were made to minimize suffering.

### Study subjects

Proteinuric patients with obesity or type 2 diabetes who underwent renal biopsy were enrolled in this study. Patients with infectious disease, cancer, liver disease or collagen disease were excluded. Proteinuria was defined as urinary protein greater than 0.5 g/g creatinine or urinary albumin greater than 300 mg/g creatinine in at least two consecutive measurements. Obesity was defined as body mass index (BMI) greater than 25.0 (kg/m^2^). Type 2 diabetes was diagnosed in accordance with the criteria of the World Health Organization. Biochemical measurements on admission for renal biopsy were used as baseline characteristics for cross-sectional analysis. Estimated glomerular filtration rate (eGFR) was calculated using a simplified prediction equation proposed by the Japanese Society of Nephrology: eGFR (ml/min/1.73 m^2^) = 194× [age (years)]^−0.287^ × [serum creatinine (mg per dl)]^−1.094^ ×0.739 (for females), which is a validated local modification of MDRD [10]. The serum concentrations of creatinine were measured using an enzymatic method.

For immunohistochemistry, 65 Japanese patients who underwent renal biopsy at Department of Medicine and Clinical Science, Kyoto University Hospital between 2000 and 2011 were analyzed. Biopsy-proven diagnoses of all patients during this period are listed in Table S1 in File S1. The subjects examined in this work included DN (n = 19), ORG (n = 10) and non-obese, non-diabetic control subjects who were diagnosed as MGA (n = 19) or MCNS (n = 17). Some cases in these categories were excluded because residual samples available contained less than 10 glomeruli. Definition of DN consisted of (1) more than 5 year duration after the onset of diabetes, (2) existence of micro- or macro-albuminuria, (3) compatible histopathological changes with DN such as glomerular basement membrane thickening, mesangial expansion, nodular sclerosis (Kimmelstiel-Wilson nodules) and/or arteriolar hyalinosis, and (4) exclusion of other causes for renal disorders [11]. ORG was defined morphologically as focal segmental glomerulosclerosis and/or glomerulomegaly in subjects having both obesity and proteinuria, whose definitions were described above [12], [13].

For mRNA expression analysis, low quality samples, in which 18S ribosomal RNA (rRNA) levels were lower than the detection sensitivity limit by real-time RT-PCR, were excluded. Subjects enrolled consisted of 22 type 2 diabetic patients who underwent renal biopsy at Osaka City General Hospital between 2000 and 2010, and 6 non-diabetic control subjects, who had biopsy-proven MGA.

Tables 1 and 2 summarize the baseline clinical characteristics of the patients who were examined by immunohistochemical or gene expression analysis, respectively. For light microscopy, the tissue specimens were processed according to standard procedures. Sections were stained with haematoxylin-eosin, periodic acid-Schiff, periodic-acid methenamine silver or Masson trichrome (Fig. S1). The ratios of the number of glomeruli with global sclerosis among that of total glomeruli and the relative areas of tubulointerstitial fibrosis were evaluated independently by two pathologists unaware of diagnosis and clinical data.

**Table 1:** Baseline clinical characteristics of patients at renal biopsy who were analyzed for MRP8 protein expression by immunohistochemistry.

	Non-obese, non-diabetic control				Between-group differences*
	MGA	MCNS	ORG	DN	
N	19	17	10	19	
Sex (male/female)	10/9	5/12	7/3	14/5	?^2^ = 8.1, P = 0.04
Age (years)	35.5±17.9	36.3±17.4	49.3±16.5	58.3±9.0	P<0.001
Diabetes duration (years)	-	-	-	11.3±6.7	-
BMI (kg/m^2^)	19.7±2.1	23.7±3.3	32.0±5.6	24.7±3.5	P<0.001
HbA1c (NGSP, %)	5.6±0.1	5.8±0.2	6.0±0.7	7.1±1.5	P = 0.04
Systolic blood pressure (mmHg)	112.2±11.4	113.0±10.2	129.3±10.4	149.4±17.2	P<0.001
Diastolic blood pressure (mmHg)	67.0±6.8	70.2±10.1	82.2±12.7	82.8±10.1	P<0.001
Urinary protein (g/g creatinine)	0.30±0.45	6.83±3.37	1.29±1.24	5.38±4.02	P<0.001
Creatinine (mg/dl)	0.66±0.14	0.68±0.15	0.84±0.23	1.45±0.66	P<0.001
eGFR (ml/min/1.73 m^2^)	100.7±24.4	94.0±20.3	75.0±18.9	45.4±19.6	P<0.001
BUN (mg/dl)	13.3±3.7	13.5±6.8	15.6±4.6	23.9±10.3	P<0.001
Total protein (g/dl)	7.0±0.6	4.7±0.8	7.0±0.5	5.8±1.0	P<0.001
Albumin (g/dl)	4.3±0.4	2.2±0.8	4.1±0.6	3.2±0.8	P<0.001
Total cholesterol (mg/dl)	184.7±38.3	442.5±123.2	206.1±27.7	247.3±50.8	P<0.001
Triglyceride (mg/dl)	100.1±84.4	230.8±140.9	124.8±63.7	175.8±75.0	P = 0.001
HDL cholesterol (mg/dl)	55.1±82.8	82.8±16.8	53.7±10.8	46.0±10.8	P<0.001
LDL cholesterol (mg/dl)	106.1±30.2	298.2±123.6	127.6±26.0	161.8±47.6	P<0.001
CRP (mg/dl)	0.3±0.9	0.3±0.8	0.3±0.2	0.1±0.2	NS
Global glomerulosclerosis (%)	2.2±4.2	2.5±4.5	22.9±15.9	33.2±17.4	P<0.001
Tubulointerstitial fibrosis (%)	0.6±1.4	0.1±0.5	15.5±9.5	38.6±18.6	P<0.001

MGA: minor glomerular abnormality, MCNS: minimal change nephrotic syndrome, ORG: obesity-related glomerulopathy, DN: diabetic nephropathy, BMI: body mass index, BUN: blood urea nitrogen, CRP: C-reactive protein. Data are means ± SD. *Overall differences between MGA, MCNS, ORG and DN groups were compared by ANOVA.

**Table 2:** Baseline clinical characteristics of patients at renal biopsy who were analyzed for MRP8 mRNA expression by real-time RT-PCR.

	MGA	DN	Between-group differences*
N	6	22	
Sex (male/female)	0/6	15/7	?^2^ = 8.8, P = 0.003
Age (years)	38.3±10.4	57.0±11.1	P = 0.001
Diabetes duration (years)	-	14.1±6.8	-
RAS blockade (yes/no)	0/0	18/4	-
BMI (kg/m^2^)	19.4±2.0	25.0±3.8	P = 0.002
HbA1c (NGSP, %)	-	7.9±2.0	-
Systolic blood pressure (mmHg)	111.2±13.5	157.9±30.1	P = 0.001
Diastolic blood pressure (mmHg)	67.3±9.0	86.8±11.7	P = 0.001
Urinary protein (g/g creatinine)	0.10±0.11	4.87±4.09	P = 0.009
Creatinine (mg/dl)	0.6±0.2	1.1±0.4	P = 0.004
eGFR (ml/min/1.73 m^2^)	92.8±26.6	57.8±27.2	P = 0.009
Total protein (g/dl)	7.0±0.4	6.2±0.9	NS
Albumin (g/dl)	4.2±0.4	3.2±0.7	P = 0.002
Total cholesterol (mg/dl)	213.5±57.2	240.1±71.2	NS
Triglyceride (mg/dl)	172.0±161.4	241.0±137.7	NS
HDL cholesterol (mg/dl)	61.3±12.5	52.5±12.0	NS
LDL cholesterol (mg/dl)	117.8±33.6	137.3±59.1	NS

RAS: renin-angiotensin system. Data are means ± SD. *Differences between MGA and DN groups were compared by unpaired t-test.

### Definition of renal outcomes

The following two prognostic indicators were examined by linear regression and logistic regression analyses, respectively: (1) the extent of proteinuria measured at one year post-biopsy, and (2) renal event defined as annual increase in serum creatinine by >50% from baseline or initiation of chronic dialysis.

### Immunohistochemistry

Immunohistochemistry of MRP8 and CD68 was carried out using kidney sections (thickness 4 µm) fixed with 4% buffered paraformaldehyde. After antigen retrieval by citrate buffer, kidney sections were incubated with 10% goat serum, followed by mouse anti-human MRP8 (1:100; BMA biomedicals, Augst, Switzerland) [14] or mouse anti-human CD68 antibodies (1∶50; DAKO, Ely, UK), respectively. Primary antibodies were visualized with horseradish peroxidase-conjugated secondary antibody and 3,3-diaminobenzidine tetrahydrochloride (Dako USA, Carpinteria, CA). Nuclei were counterstained with hematoxylin. MRP8-positive cells were counted in more than 10 glomeruli, and MRP8-positive area in tubulointerstitium was measured quantitatively to obtain an average for each subject using MetaMorph 7.5 software (Molecular Devices, Downingtown, PA, USA). Co-localization of CD68- and MRP8-positive cells was evaluated with serial sections. There was neither MRP8 nor CD68 signal in negative controls stained without first antibody (Fig. S2). By preincubation of anti-MRP8 antibody with 20 molar excess of recombinant human MRP8 protein (Life Technologies, Carlsbad, CA, USA) at 4°C overnight, staining was markedly reduced, if not completely, further supporting the specificity of the antibody (Fig. S3).

### Evaluation of mRNA expression

Frozen kidney sections were separated into glomeruli and non-glomerulus tissues by laser capture micro-dissection (LM200; Olympus, Tokyo, Japan) as previously described [15]. Total RNA was extracted with RNeasy mini kit (Qiagen, Tokyo, Japan). mRNA expression levels were determined by TaqMan real-time PCR (Applied Biosystems, Foster City, CA, USA) [16], [17]. Expression levels of all genes were normalized by 18S rRNA (internal control) levels. See Table S2 in File S1 for primer and probe sequences. Eukaryotic 18S rRNA was detected with Pre-Developed TaqMan Assay Reagents (Applied Biosystems).

### MRP8 treatment of macrophages

Bone marrow-derived macrophages were generated from wild-type or TLR4 knockout (KO) mice [18] on C57BL/6J genetic background (Oriental BioService, Kyoto, Japan) as described previously [6]. Briefly, following lysis of red blood cells, bone marrow cells were resuspended in medium containing 20% fetal calf serum and 50 ng/ml recombinant human macrophage colony-stimulating factor (Peprotech, Rocky Hill, NJ, USA), and cultured at 37°C in 5% CO_2_ atmosphere. On day 7, macrophages were incubated with recombinant mouse MRP8 (Abnova, Taipei, Taiwan) or vehicle for 4 hours. Polymyxin B (25 µg/ml, Nacalai Tesque, Kyoto, Japan) was added to each well to minimize contamination of endotoxin as described previously [3], [19]. No endotoxin was detected at any concentration of MRP8 tested here after incubation with 25 µg/ml of polymyxin B by ToxinSensor Chromogenic LAL Endotoxin Assay Kit (GenScript, Piscataway, NJ, USA). Total RNA from cells was extracted with RNeasy Mini Kit, and mRNA expression levels of interleukin-1 beta (IL-1β), tumor necrosis factor alpha (TNFα) and MRP8 were determined by TaqMan real-time RT-PCR. Expression levels of all genes were normalized by rodent GAPDH levels (internal control, Pre-Developed TaqMan Assay Reagents). Primer and Probe sequences for real-time PCR are listed in Table S2 in File S1.

### Statistical analysis

Data are expressed as means ± SD, or means ±95% confidence interval (CI) when appropriate. For the comparison among four groups, one-way or two-way ANOVA with Bonferroni' post-hoc analysis was used, and categorical variables were compared using χ^2^ test. Student's unpaired t-test was applied for comparison between two groups as appropriate. Spearman's correlation coefficients were estimated to determine associations between two variables. To examine the effects of baseline covariates determining the extent of glomerular or tubulointerstitial MRP8 expression or urinary protein levels one year after biopsy, univariate and multivariate linear regression analyses were performed. Logistic regression analysis was used to analyze explanatory variables predicting the occurrence of renal event. All data were analyzed using StatView 5.0 software (SAS Institute Inc., Cary, NC, USA). P values <0.05 were considered statistically significant.

## Results

**Figure 1:**
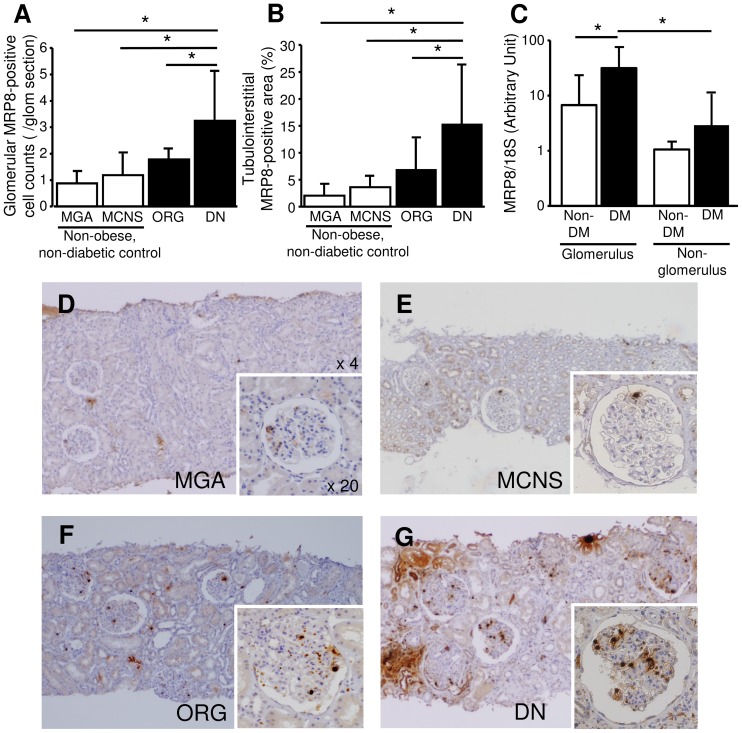
Immunohistochemical and mRNA analyses for MRP8 in kidney biopsy samples. Quantification of glomerular MRP8-positive cell count (A) and tubulointerstitial MRP8-positive area (B). mRNA expression of MRP8 in glomerular and non-glomerular fractions (C). Open bars: non-obese, non-diabetic controls which are MGA or MCNS, closed bars: ORG or DN (A–C). Representative pictures of MGA, MCNS, ORG and DN groups (D–G). MGA: minor glomerular abnormality, MCNS: minimal change nephrotic syndrome, ORG: obesity-related glomerulopathy, DN: diabetic nephropathy. *P<0.01.

**Figure 2:**
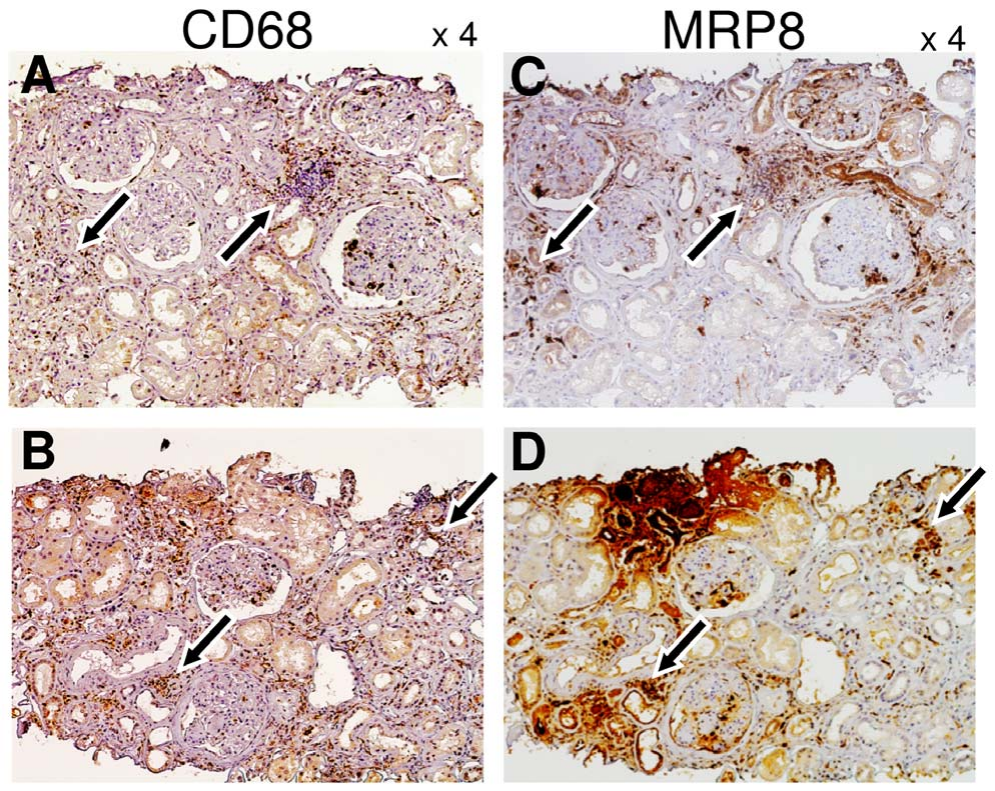
Localization of CD68 and MRP8 protein expression in serial sections of diabetic nephropathy cases. Expression of CD68 (A, B) and MRP8 expression (C, D) in paired renal specimens (A and C, or B and D). Arrows indicate colocalization of CD68 and MRP8 signals.

We compared MRP8 protein expression levels in the kidneys among DN, ORG and non-obese, non-diabetic control (MGA and MCNS) groups. Immunohistochemical analysis revealed that both glomerular MRP8-positive cell count (Fig. 1A) and tubulointerstitial MRP8-positive area (Fig. 1B) in DN were significantly larger than those in other groups including MGA, MCNS and ORG (P<0.01). ORG subjects also showed a tendency of elevated MRP8 expression compared to MGA and MCNS (Fig. 1A, 1B). Furthermore, glomerular MRP8 mRNA expression levels in DN subjects were significantly higher compared to non-DM control subjects (P<0.01, Fig. 1C). In non-glomerulus tissues, MRP8 mRNA expression levels were much lower than those in glomeruli, both in non-DM and DM groups. Abundant MRP8 protein expression in the tubulointerstitium of DN cases was not clearly reflected into increased mRNA expression, which may be partly caused by deposition of blood-derived proteins in the tubulointerstitium as discussed in the next section. As shown in representative photos (Fig. 1D–G, see Fig. S4 in detail), renal biopsy samples from MGA and MCNS subjects showed few MRP8-positive cells in glomeruli (Fig. 1D, 1E and Fig. S4). In ORG subjects, some MRP8-postive cells appeared in glomeruli and tubulointerstitium (Fig. 1F and Fig. S4). In DN subjects, marked increase of MRP8-expressing cells in glomeruli and significant expansion of MRP8-positive areas in the tubulointerstitium were observed in a focal manner (Fig. 1G and Fig. S4). Of note, MRP8-positive cells were absent in nodular sclerosing lesions of diabetic glomeruli (Fig. S4: DN case 2, 3) as described previously for sclerotic lesions in ANCA-associated glomerulonephritis [20]. Paired immunohistochemistry for CD68 and MRP8 in serial sections suggested that MRP8 signals were, at least in part, observed in macrophages expressing CD68 (Fig. 2), as we reported in a mouse model of diabetic nephropathy [6]. Besides, focally injured atrophic tubular epithelial cells also strongly expressed MRP8, which were surrounded by MRP8(+)-, CD68(+)-positive macrophages (Fig. 2, Fig S4: DN case 3-5). In the cases with nephrotic range proteinuria, MRP8 staining was also observed along brush borders of proximal tubules both in MCNS and DN cases (Fig. S4). Since sample number of mRNA expression was too small for multivariate analysis, the following analyses were performed using data from patients studied by immunohistochemistry.

The associations between kidney MRP8 signals and baseline clinical parameters at the time of renal biopsy were analyzed cross-sectionally (Table 3). By univariate analysis, glomerular and/or tubulointerstitial MRP8 protein expression was significantly correlated to age, systolic and diastolic blood pressures, urinary protein, serum levels of creatinine, BUN and HDL cholesterol, eGFR, and extent of global glomerulosclerosis and tubulointerstitial fibrosis. These parameters were further examined by multivariate analysis after excluding diastolic blood pressure, eGFR and BUN because of collinearity. Percentage of tubulointerstitial fibrosis was independently correlated with glomerular MRP8 signals (β = 0.62, adjusted P = 0.02) and tubulointerstitial MRP8 signals (β = 0.85, adjusted P<0.001), respectively. Additionally, tubulointerstitial MRP8 signals were also independently correlated with baseline proteinuria (β = 0.20, adjusted P = 0.01).

**Table 3:** Relationship between baseline clinical parameters and MRP8 signals.

	Glomerular MRP8-positive cell count				Tubulointerstitial MRP8-positive area			
	Univariate		Multivariate*		Univariate		Multivariate^#^	
	β	P	β	P	β	P	β	P
Sex (male)	0.21	0.09			0.16	0.20		
Age (y)	0.37	0.002	0.02	0.87	0.39	0.001	0.03	0.74
Diabetes duration (y)	0.28	0.14			0.33	0.08		
BMI (kg/m^2^)	0.14	0.27			0.09	0.48		
HbA1c (NGSP, %)	0.11	0.52			0.04	0.82		
Systolic BP (mmHg)	0.62	<0.001	0.18	0.29	0.64	<0.001	−0.12	0.37
Diastolic BP (mmHg)	0.43	<0.001			0.42	<0.001		
Urinary protein (g/gCr)	0.37	0.003	0.18	0.07	0.43	<0.001	0.20	0.01
Creatinine (mg/dl)	0.60	<0.001	0.01	0.10	0.75	<0.001	0.20	0.08
eGFR (ml/min/1.73 m^2^)	−0.49	<0.001			−0.70	<0.001		
BUN (mg/dl)	0.56	<0.001			0.71	<0.001		
Total protein (g/dl)	−0.16	0.21			−0.13	0.30		
Albumin (g/dl)	−0.22	0.09			−0.21	0.09		
T-chol (mg/dl)	−0.06	0.66			−0.04	0.76		
Triglyceride (mg/dl)	0.02	0.84			0.11	0.38		
HDL-chol (mg/dl)	−0.24	0.06			−0.28	0.03	−0.03	0.73
LDL-chol (mg/dl)	0.00	0.98			0.00	0.99		
CRP (mg/dl)	0.03	0.79			0.12	0.34		
Global GS (%)	0.52	<0.001	−0.17	0.40	0.61	<0.001	−0.27	0.07
TI fibrosis (%)	0.68	<0.001	0.62	0.02	0.80	<0.001	0.85	<0.001

Coefficient of determination (R^2^) calculated with explanatory parameters enrolled in multiple regression analysis was 0.52* and 0.74^#^, respectively. y, years; BP, blood pressure; gCr, g creatinine; T-chol, total cholesterol; HDL-chol, HDL cholesterol; LDL-chol, LDL cholesterol; GS, glomerulosclerosis; TI, tubulointerstitial.

Scattered plot analyses between MRP8 signals in glomeruli or tubulointerstitium and clinical parameters indicated that MCNS group had a distinct distribution pattern from other groups, especially as to urinary protein and serum LDL cholesterol levels (Fig. S5A–D, S6A–D). Exclusion of MCNS group improved correlation between MRP8 signals and urinary protein or serum LDL-cholesterol levels (Fig. S5E–F, S6E–F). Therefore, we carried out sub-analysis excluding MCNS patients, and found that urinary protein was an independent factor correlated with glomerular MRP8 signals by multivariate analysis (Table 4; β = 0.36, adjusted P = 0.03).

**Table 4:** Sub-analysis of relationship between baseline clinical parameters and MRP8 signals, after exclusion of MCNS group.

	Glomerular MRP8(+) cell count				Tubulointerstitial MRP8(+) area			
	Univariate		Multivariate*		Univariate		Multivariate^#^	
	β	P	β	P	β	P	β	P
Systolic BP (mmHg)	0.64	<0.001	0.16	0.38	0.63	<0.001	−0.16	0.23
Urinary protein (g/gCr)	0.70	<0.001	0.36	0.03	0.75	<0.001	0.47	<0.001
Creatinine (mg/dl)	0.62	<0.001	0.05	0.80	0.75	<0.001	0.24	0.09
Total protein (g/dl)	−0.51	<0.001	0.03	0.84	−0.49	<0.001	0.12	0.25
Triglyceride (mg/dl)	0.31	0.003			0.37	0.01		
HDL-chol (mg/dl)	−0.16	0.27			−0.20	0.18		
LDL-chol (mg/dl)	0.60	<0.001	0.13	0.35	0.52	0.001	−0.10	0.35
Global GS (%)	0.49	<0.001	−0.20	0.29	0.59	<0.001	−0.17	0.23
TI fibrosis (%)	0.68	<0.001	0.38	0.18	0.80	<0.001	0.70	0.002

R^2^ was 0.60* and 0.77^#^, respectively. BP, blood pressure; gCr, g creatinine; HDL-chol, HDL cholesterol; LDL-chol, LDL cholesterol; GS, glomerulosclerosis; TI, tubulointerstitial.

Next, we performed linear regression or logistic regression analyses to identify explanatory factors predicting renal outcomes which were extent of proteinuria a year later and renal event within a year. Since there was a good association between glomerular and tubulointerstitial MRP8 signals (Fig. S7; R = 0.67, P<0.001), these parameters were alternatively enrolled in further analyses. We evaluated association between baseline parameters and urinary protein at 1 year after renal biopsy by multiple regression analysis. As shown in Table 5, glomerular MRP8 signal (β = 0.59, adjusted P<0.001) was a predictive factor for the extent of proteinuria a year later, as well as baseline systolic blood pressure (β = 0.21, adjusted P = 0.04) and baseline proteinuria (β = 0.37, adjusted P = 0.002) were. These parameters were independent from other known diabetic nephropathy risk factors including renal dysfunction (serum creatinine) and extent of global sclerosis and tubulointerstitial fibrosis [11], [21]–[24]. On the other hand, tubulointerstitial MRP8 signal (β = 0.34, adjusted P = 0.09) was not an independent predictive factor for urinary protein levels a year later. Renal events occurred in 7 patients (6 in DN and 1 in ORG cases) within a year after renal biopsy. By univariate analysis, not only extent of glomerulosclerosis and tubulointerstitial fibrosis, and glomerular and tubulointerstitial MRP8 signals, but also blood pressures, renal dysfunction and urinary protein levels at baseline were significant predictive factors for the occurrence of renal events. However, by multivariate analysis, there covariates were cancelled out by each other (Table S3 in File S1), likely due to high correlations among these parameters.

**Table 5:** Multiple regression analysis for identification of factors predicting urinary protein levels 1 year after renal biopsy.

	Urinary protein 1-year after renal biopsy					
	Univariate		Multivariate			
			Model 1*		Model 2^#^	
	β	P	β	P	β	P
Sex (male)	−0.14	0.50				
Age (y)	−0.01	0.95				
Diabetes duration (y)	0.24	0.22				
BMI (kg/m^2)^	−0.33	0.10				
HbA1c (NGSP, %)	0.08	0.70				
Systolic BP (mmHg)	0.43	0.03	0.21	0.04	0.30	0.10
Diastolic BP (mmHg)	0.07	0.73				
Urinary protein (g/gCr)	0.78	<0.001	0.37	0.002	0.55	0.002
Creatinine (mg/dl)	0.44	0.02				
eGFR (ml/min/1.73 m^2^)	−0.56	0.003	−0.31	0.76	0.18	0.45
BUN (mg/dl)	0.46	0.02				
Total protein (g/dl)	−0.54	0.004				
Albumin (g/dl)	−0.69	<0.001				
T-chol (mg/dl)	0.66	<0.001				
Triglyceride (mg/dl)	0.18	0.38				
HDL-chol (mg/dl)	−0.02	0.91				
LDL-chol (mg/dl)	0.62	<0.001				
CRP (mg/dl)	−0.11	0.57				
Global glomerulosclerosis (%)	−0.05	0.79	−0.17	0.11	−0.31	0.06
Tubulointerstitial fibrosis (%)	0.43	0.02	−0.02	0.91	0.10	0.67
Glomerular MRP8(+) cell count	0.87	<0.001	0.59	<0.001		
Tubulointerstitial MRP8(+) area (%)	0.67	0.001			0.34	0.09

R^2^ was 0.91* and 0.75^#^, respectively. y, years; BP, blood pressure; gCr, g creatinine;

T-chol, total cholesterol; HDL-chol, HDL cholesterol; LDL-chol, LDL cholesterol.

**Figure 3:**
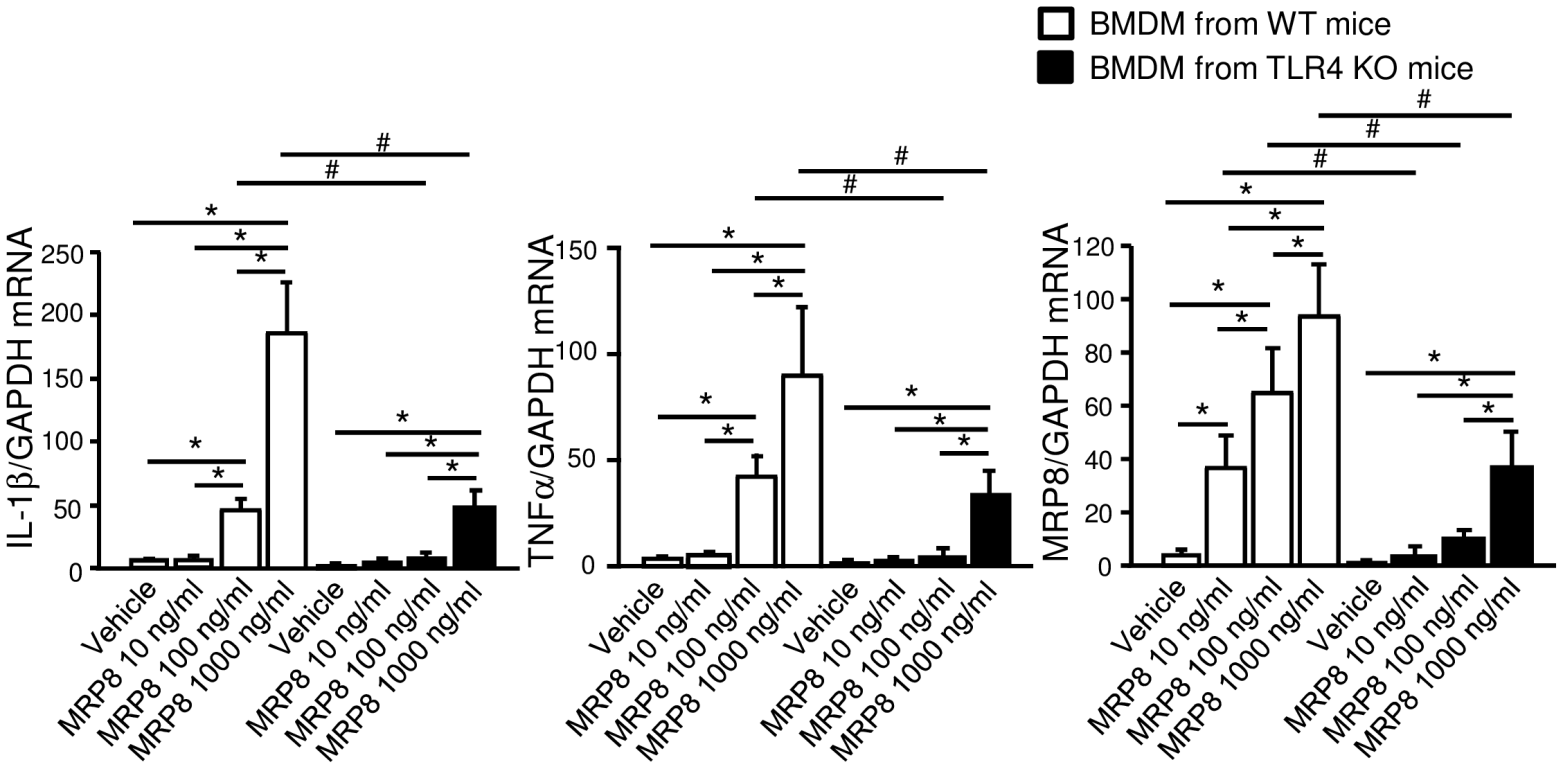
Effects of MRP8 upon bone marrow-derived macrophages. Bone marrow-derived macrophages (BMDM) were stimulated with recombinant mouse MRP8 for 4 hours. Error bars indicate 95% CI and statistical analyses were performed with log-transformed values. Two-way ANOVA revealed significant effects of genotypes, MRP8 concentrations and their interactions for expression of all 3 genes (P<0.001 for all comparisons). n = 4. WT, wild-type; KO, knockout; IL-1β, interleukin 1 beta; TNFα, tumor necrosis factor alpha. *P<0.01 among different concentrations, ^#^P<0.01 among genotypes.

Finally, we examined the potency of MRP8 as an endogenous ligand for TLR4 using cultured macrophages. In bone marrow-derived macrophages from wild-type mice, MRP8 protein induced upregulation of proinflammatory cytokine genes such as IL-1β and TNFα and also triggered auto-induction of MRP8, in a dose-dependent manner between 10–1000 ng/ml. These effects of MRP8 were suppressed approximately by two-thirds in macrophages obtained from TLR4 KO mice (P<0.01) (Fig. 3).

## Discussion

The present study has demonstrated that MRP8 is abundantly expressed in glomeruli and tubulointerstitium of patients with DN as compared to ORG and non-obese, non-diabetic control (MGA and MCNS). Furthermore, in ORG subjects, MRP8 expression levels tended to be higher than MGA or MCNS subjects. In baseline cross-sectional investigation including all subjects, by univariate linear regression analysis, glomerular MRP8-positive cell count and tubulointerstitial MRP8-positive area were both, respectively, correlated not only with various known risk factors for diabetic nephropathy (such as systolic blood pressure, proteinuria and serum creatinine) but also with extent of glomerulosclerosis and tubulointerstitial fibrosis. By multivariate analysis, tubulointerstitial MRP8-positive area was significantly correlated with proteinuria and tubulointerstitial fibrosis. Glomerular MRP8-positive cell count was significantly correlated with tubulointerstitial fibrosis in the primary analysis, and with proteinuria in a sub-analysis excluding MCNS group. Immunohistochemistry indicated that MRP8 was expressed, at least partly, by CD68(+)-expressing macrophages and atrophic tubules. These findings raise a possibility that kidney MRP8 signals in glomeruli or tubulointerstitium may serve as novel markers of diabetic nephropathy.

In prognostic study, multivariate analysis revealed that urinary protein levels at a year after renal biopsy were independently associated with glomerular MRP8-positive cell count, urinary protein and systolic blood pressure at baseline. Of note, glomerular MRP8 expression showed the strongest correlation with urinary protein a year later (β = 0.87), even stronger than baseline urinary protein (β = 0.78), by univariate analysis. It is partly because glomerular MRP8 expression is not largely elevated in ‘benign’ forms of proteinuria such as ones observed in MCNS patients, whose levels of proteinuria are extremely high at diagnosis by renal biopsy but are usually resolved within a year after initiation of immunosuppressive therapy. These findings suggest that glomerular MRP8 expression may possess a unique predictive nature as a disease marker, which cannot be substituted by baseline proteinuria or routine pathological analysis evaluating global glomerulosclerosis and tubulointerstitial fibrosis. Moreover, we speculate that glomerular MRP8 expression is not a simple marker or bystander but an active player in glomerular injury as discussed below.

In another attempt of longitudinal study, logistic regression analysis failed to find out any independent predictors for the occurrence of renal event within a year. We speculate that this is partly because tubulointerstitial MRP8 expression and tubulointerstitial fibrosis were two potent predictors for renal event in univariate analysis but their significances were canceled by each other in multivariate analysis. These two parameters showed strong correlation (R = 0.68, P<0.001) (Fig. S8), suggesting that these two parameters might be equivalent to predict renal event. Indeed, interstitial MRP8 expression showed quite similar pattern to interstitial fibrosis evaluated by Masson trichrome staining. Quantity of interstitial MRP8 largely depends on the positive signals in atrophic tubules rather than those in macrophages, whose feature differs from that of glomerular MRP8 in punctate distribution. Furthermore, small sample size, short observation period and few subjects who developed renal event might have reduced the detection power. Since MRP8 expression in tubular epithelial cells plays a causative role in the progression of tubulointerstitial inflammation in a mouse model of renal fibrosis [7], further analysis will be needed to clarify the role of tubulointerstitial MRP8 in DN.

In accordance with our previous study [6], MRP8 mRNA was upregulated predominantly in the glomerular fraction of human DN subjects as compared to control subjects with MGA. On the other hand, MRP8 protein expression was observed not only in the glomerulus but also in the tubulointerstitium. In this regard, it should be noted that there were two distinct patterns of MRP8 staining in the tubulointerstitium of DN. One was intense and focal staining in severely atrophic tubules. The other was mild staining distributed along the brush border of proximal tubules, which was also found in ORG and MCNS. The latter signals likely represent MRP8 protein derived from the blood and reabsorbed by proximal tubules, which should not be accompanied by increased MRP8 mRNA expression. Concerning proteins other than MRP8, we and others have recently reported similar phenomena of immunoreactive protein detection in the proximal tubules caused by reabsorption but not by renal synthesis [25], [26]. On the other hand, since a little MRP8 staining remained in antibody absorption test, especially in glomerular exudative lesions and severely-scarred, fibrotic lesions around atrophic tubules, presence of non-specific signals cannot be completely negated (Fig. S3).

Glomerular MRP8 signals mainly showed punctuate pattern in DN subjects (Fig. 1, Fig. S4). Since both of CD68 and MRP8 were detected by mouse monoclonal antibodies, localization of these molecules were evaluated by serial sections, not by double-immunostaining. Staining patterns of MRP8 were compatible with those in other inflammatory renal disorders including IgA nephritis [27], membrano-proliferative glomerulonephritis [14], and ANCA-related glomerulonephritis [20], in which macrophages were suggested as a major source of MRP8, as we reported in a rodent model [6]. In addition, neutrophils could be considered as another source of MRP8 affecting vascular complications [28]. Currently, we are investigating the molecular mechanism why MRP8 is predominantly upregulated in myeloid-lineage cells infiltrating glomeruli. In vitro study revealed that MRP8 induced inflammatory cytokine expression and also potentiated expression of MRP8 itself in macrophages in a TLR4-dependent manner. Additionally, MRP8-positive cells were absent in nodular sclerosing lesion, suggesting that glomerular MRP8 might reflect on-going glomerular damage [20]. Importantly, a largest-scale human study reported that MRP8 gene expression in blood mononuclear cells of type 1 diabetic patients is significantly elevated in subjects with diabetic complications including nephropathy [29].

Since inhibition of renin-angiotensin system (RAS) is an important determinant of renal outcomes, we examined the effects of RAS blockade on kidney MRP8 expression. We found no significant difference in kidney MRP8 mRNA expression between DN patients treated with or without RAS blockade (Fig. S9), probably because cases treated with RAS blockade tended to have more severe hypertension and proteinuria than cases without RAS blockade.

In obese humans and mice, increased plasma MRP8/14 complex may reflect a degree of obesity and originate from adipocytes as well as leukocytes [8], [9]. Our ORG cases had mild staining of MRP8 in proximal tubules, suggesting increased plasma levels of MRP8. In contrast, there was no significant correlation between tubulointerstitial MRP8 expression and body mass index (Fig. S6G). Therefore, local MRP8 expression in the kidney may serve better as a marker for renal injury rather than for obesity [8], [9].

Our study has several limitations. Sample number of each group studied was small. Non-identical subjects were enrolled in the mRNA and immunohistochemical analyses. Since we only analyzed patients who underwent renal biopsy, the composition of patients investigated here may not reflect those in general type 2 diabetic patients or in general chronic kidney disease subjects. Although age was not retained as an independent factor associated with MRP8 signals in our data (Table 3), it is known that aging associates chronic inflammation [30]. The effects of age cannot be completely neglected. Although most MRP8 signals were lost in antibody absorption test, there were some positive signals remaining, which might be caused by a non-specific binding of the first antibody. As discussed above, investigation of renal MRP8 expression by renal biopsy helps us understand the pathophysiology and prognosis of chronic kidney diseases, especially associated with obesity and diabetes, but has a disadvantage for routine and repeated use in out-patient clinics.

In summary, the present study suggests that expression of MRP8 in the kidney reflects the current pathological status and also predicts renal outcomes in patients with obesity or type 2 diabetes. Further investigations studying urinary MRP8 levels among obese or diabetic patients in large scale may be warranted.

## Supporting Information

Figure S1 **Representative photos showing renal biopsy sections of a DN patient stained with (A) periodic acid-Schiff, (B) periodic-acid methenamine silver or (C) Masson trichrome.** The ratio of the number of glomeruli with global sclerosis (arrows) among that of total glomeruli and the relative area of tubulointerstitial fibrosis were 33% and 65%, respectively, in this patient.
(TIFF)

Figure S2 **Immunohistochemistry for MRP8 and CD68 proteins in DN patients.** Photos in the right column show negative control experiments without 1^st^ antibody.
(TIF)

Figure S3 **Antibody absorption test for MRP8 staining.** PBS: phosphate buffered saline, rhMRP8: recombinant human MRP8.
(TIF)

Figure S4 **Representative photos of MRP8 expression in MGA, MCNS, ORG and DN groups.**
(TIF)

Figure S5 **Correlation between glomerular MRP8-positive cell count and clinical parameters.** The log-transformed values of MRP8 signals were used. The correlations were analyzed using all of 4 groups (A–D, G) or 3 groups excluding MCNS group (E, F). Open circles: minor glomerular abnormality (MGA), closed circles: minimal change nephrotic syndrome (MCNS), open triangles: obesity-related glomerulopathy (ORG), closed triangles: diabetic nephropathy (DN).
(TIF)

Figure S6 **Correlation between tubulointerstitial MRP8-positive area and clinical parameters.** The log-transformed values of MRP8 signals were used. These correlations were analyzed using all of the 4 groups (A–D, G) or 3 groups excluding MCNS group (E, F).
(TIF)

Figure S7 **Correlation between glomerular and tubulointerstitial MRP8 expression.** The log-transformed values of MRP8 signals were used.
(TIF)

Figure S8 **Correlation between tubulointerstitial MRP8-positive area and tubulointerstitial fibrosis.** The log-transformed values of MRP8 signals were used.
(TIF)

Figure S9 **Renal mRNA expression of MRP8 in DN patients with or without renin-angiotensin blockade.** N.S.: not significant. n = 15 (Yes), 6 (No). Among 22 DN cases, information about medication was not available in one patient.
(TIF)

File S1 **Supporting Tables.** Table S1, Pathological diagnoses of all cases who underwent renal biopsy at Department of Medicine and Clinical Science, Kyoto University Hospital between 2000 and 2011. Table S2, Primer and probe sequences for TaqMan real-time RT-PCR. Table S3, Logistic regression analysis for the occurrence of renal event within a year.
(DOC)
