# Supplementary figures and images for: Predictive Significance of Kidney Myeloid-Related Protein 8 Expression in Patients with Obesity- or Type 2 Diabetes-Associated Kidney Diseases
Source: PLoS One. 2014 Feb 18;9(2):e88942. doi: 10.1371/journal.pone.0088942 (PMC3928329; doi:10.1371/journal.pone.0088942)

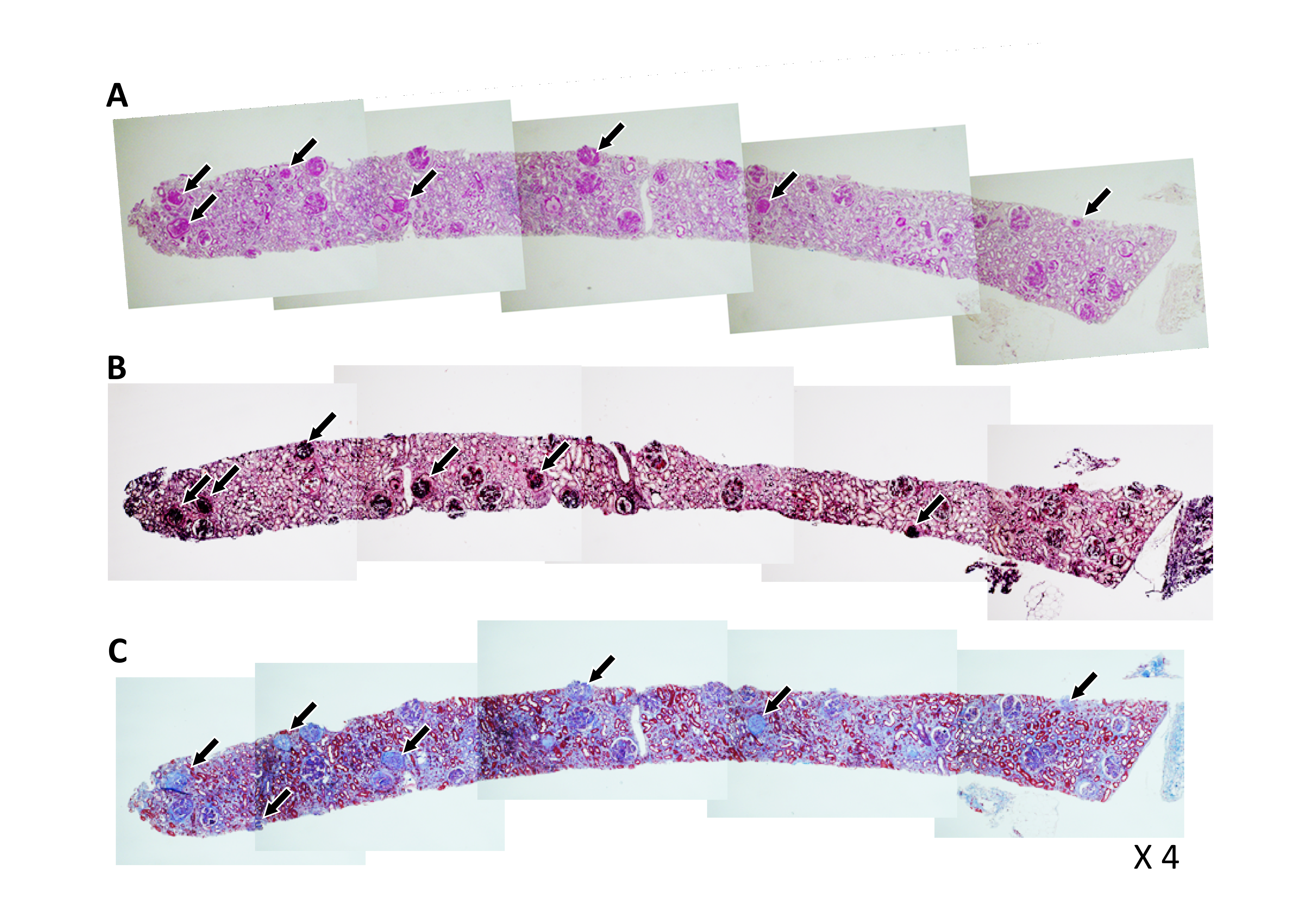

Supplement: Figure S1 — Representative photos showing renal biopsy sections of a DN patient stained with (A) periodic acid-Schiff, (B) periodic-acid methenamine silver or (C) Masson trichrome. The ratio of the number of glomeruli with global sclerosis (arrows) among that of total glomeruli and the relative area of tubulointerstitial fibrosis were 33% and 65%, respectively, in this patient. (TIFF) [file pone.0088942.s001.tiff]

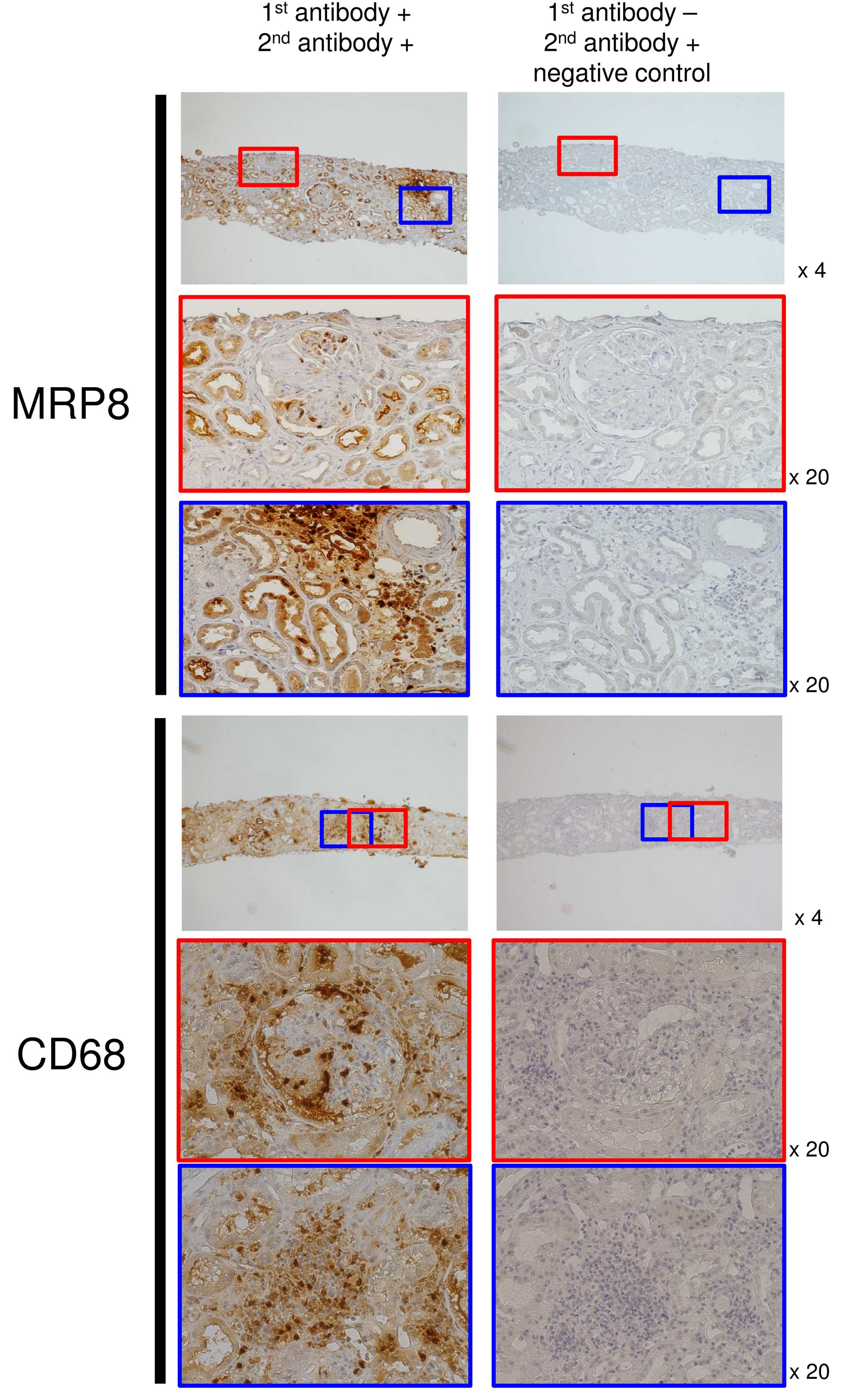

Supplement: Figure S2 — Immunohistochemistry for MRP8 and CD68 proteins in DN patients. Photos in the right column show negative control experiments without 1st antibody. (TIF) [file pone.0088942.s002.tif]

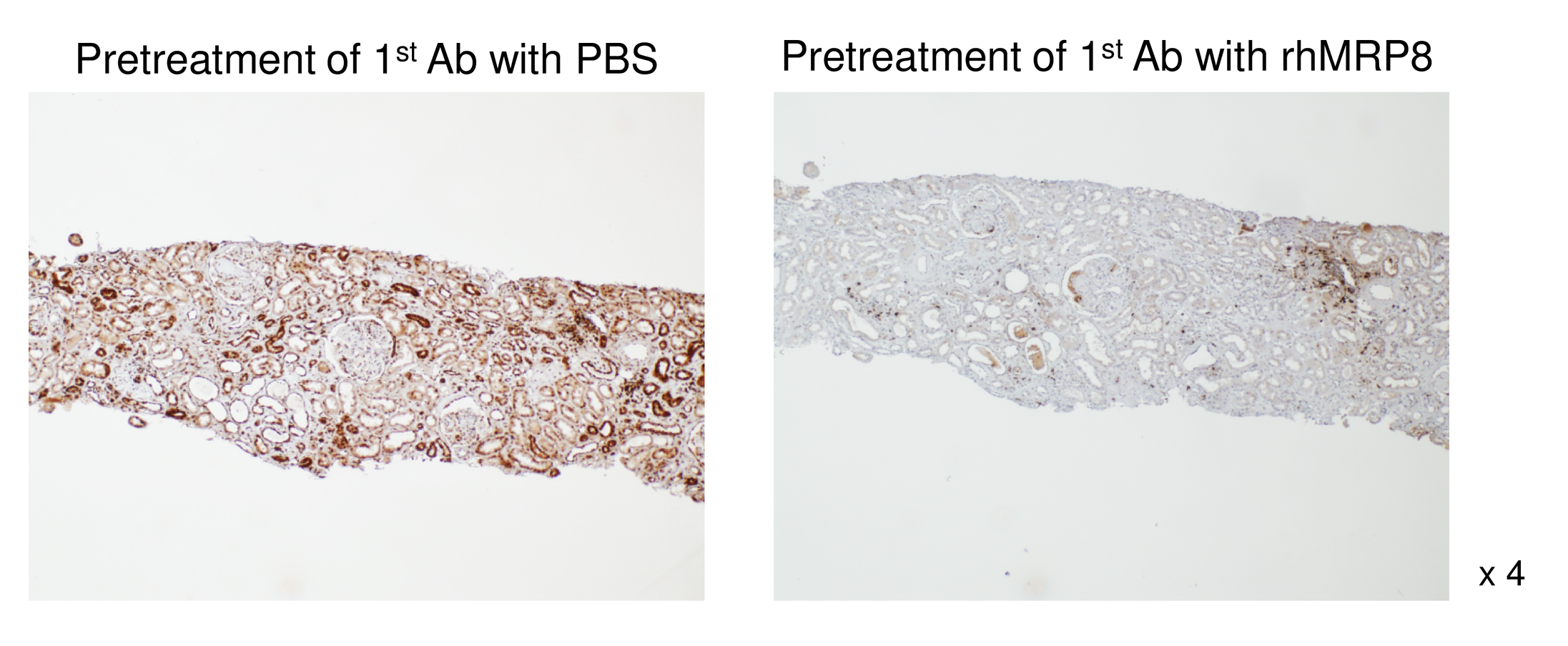

Supplement: Figure S3 — Antibody absorption test for MRP8 staining. PBS: phosphate buffered saline, rhMRP8: recombinant human MRP8. (TIF) [file pone.0088942.s003.tif]

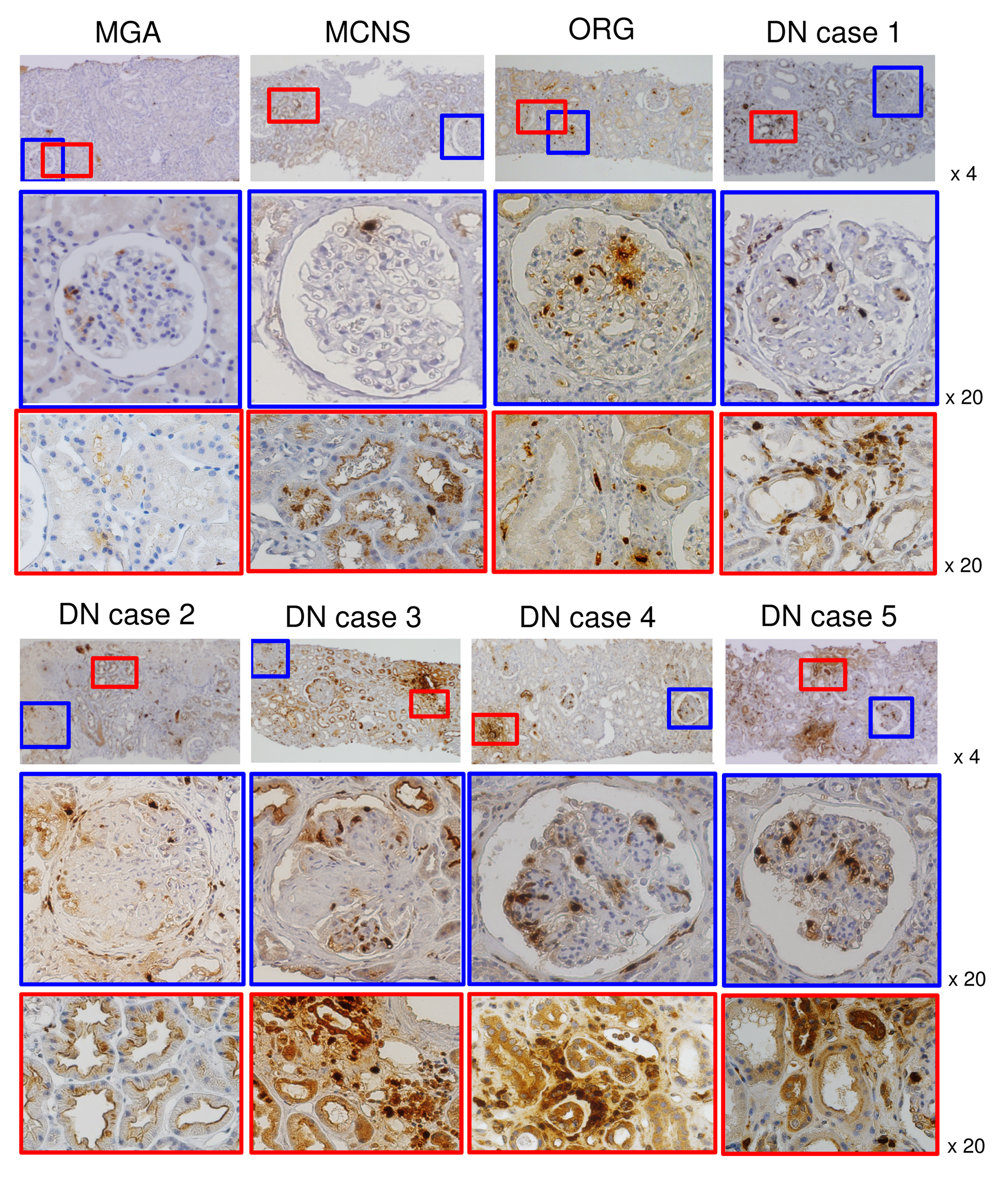

Supplement: Figure S4 — Representative photos of MRP8 expression in MGA, MCNS, ORG and DN groups. (TIF) [file pone.0088942.s004.tif]

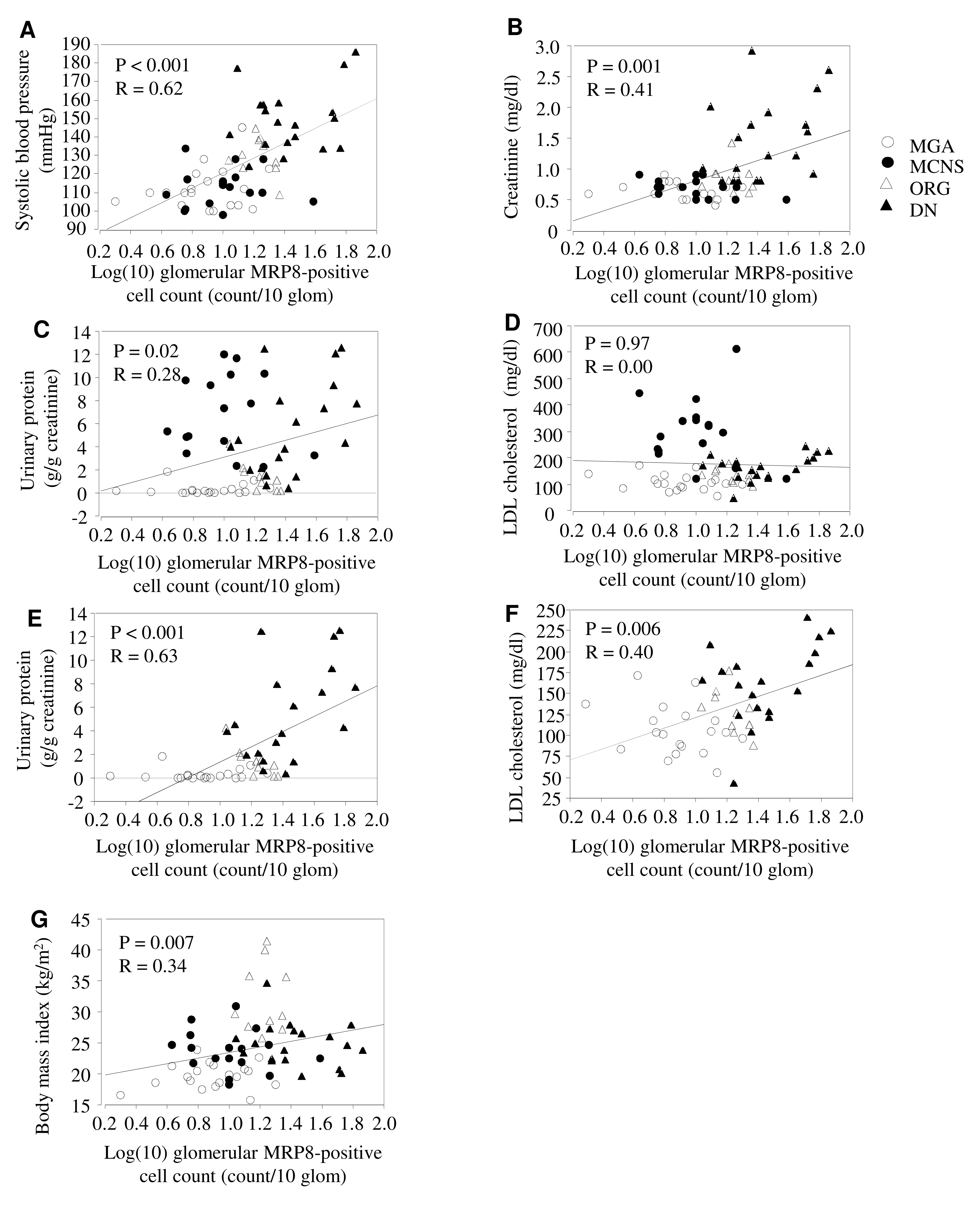

Supplement: Figure S5 — Correlation between glomerular MRP8-positive cell count and clinical parameters. The log-transformed values of MRP8 signals were used. The correlations were analyzed using all of 4 groups (A–D, G) or 3 groups excluding MCNS group (E, F). Open circles: minor glomerular abnormality (MGA), closed circles: minimal change nephrotic syndrome (MCNS), open triangles: obesity-related glomerulopathy (ORG), closed triangles: diabetic nephropathy (DN). (TIF) [file pone.0088942.s005.tif]

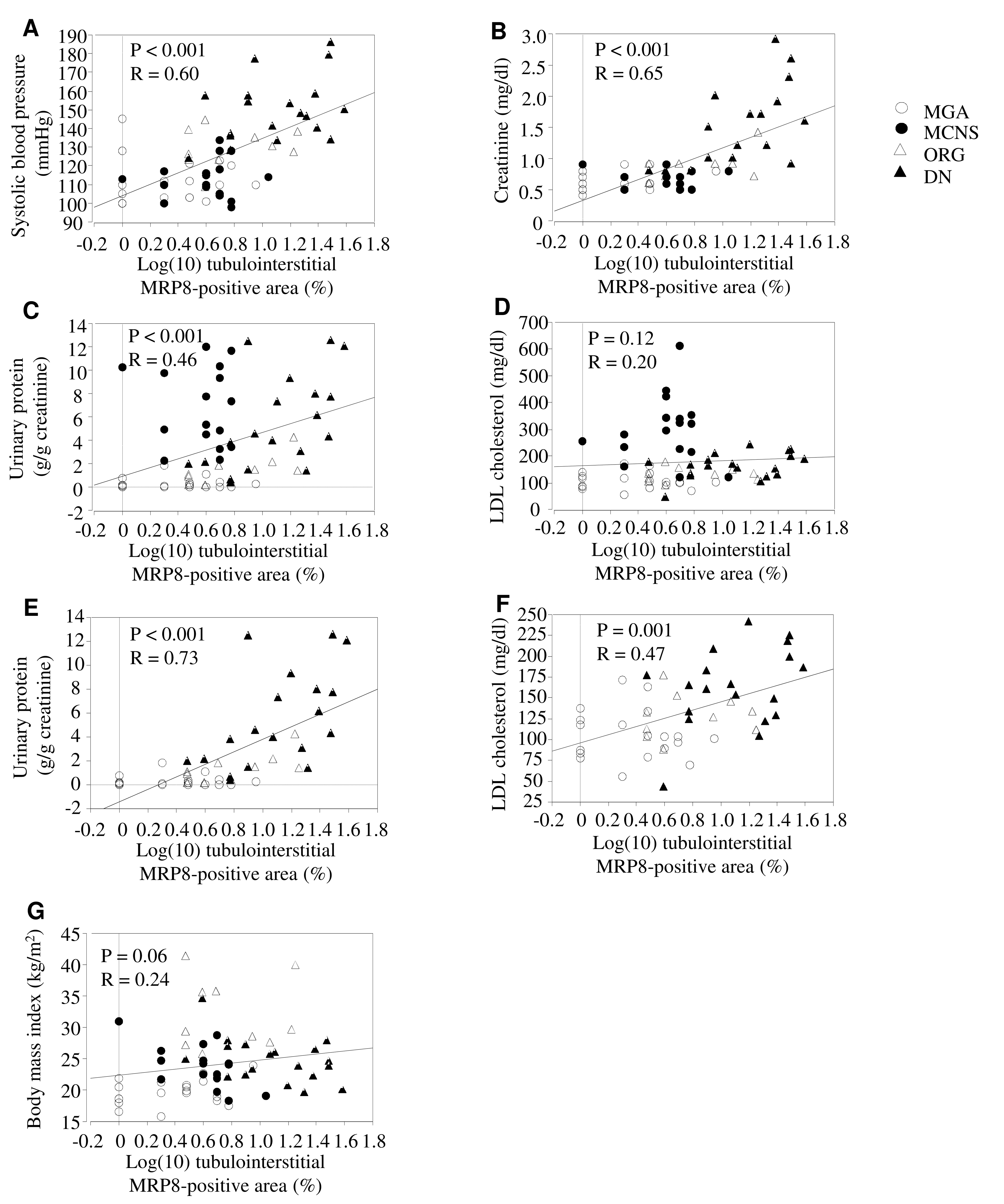

Supplement: Figure S6 — Correlation between tubulointerstitial MRP8-positive area and clinical parameters. The log-transformed values of MRP8 signals were used. These correlations were analyzed using all of the 4 groups (A–D, G) or 3 groups excluding MCNS group (E, F). (TIF) [file pone.0088942.s006.tif]

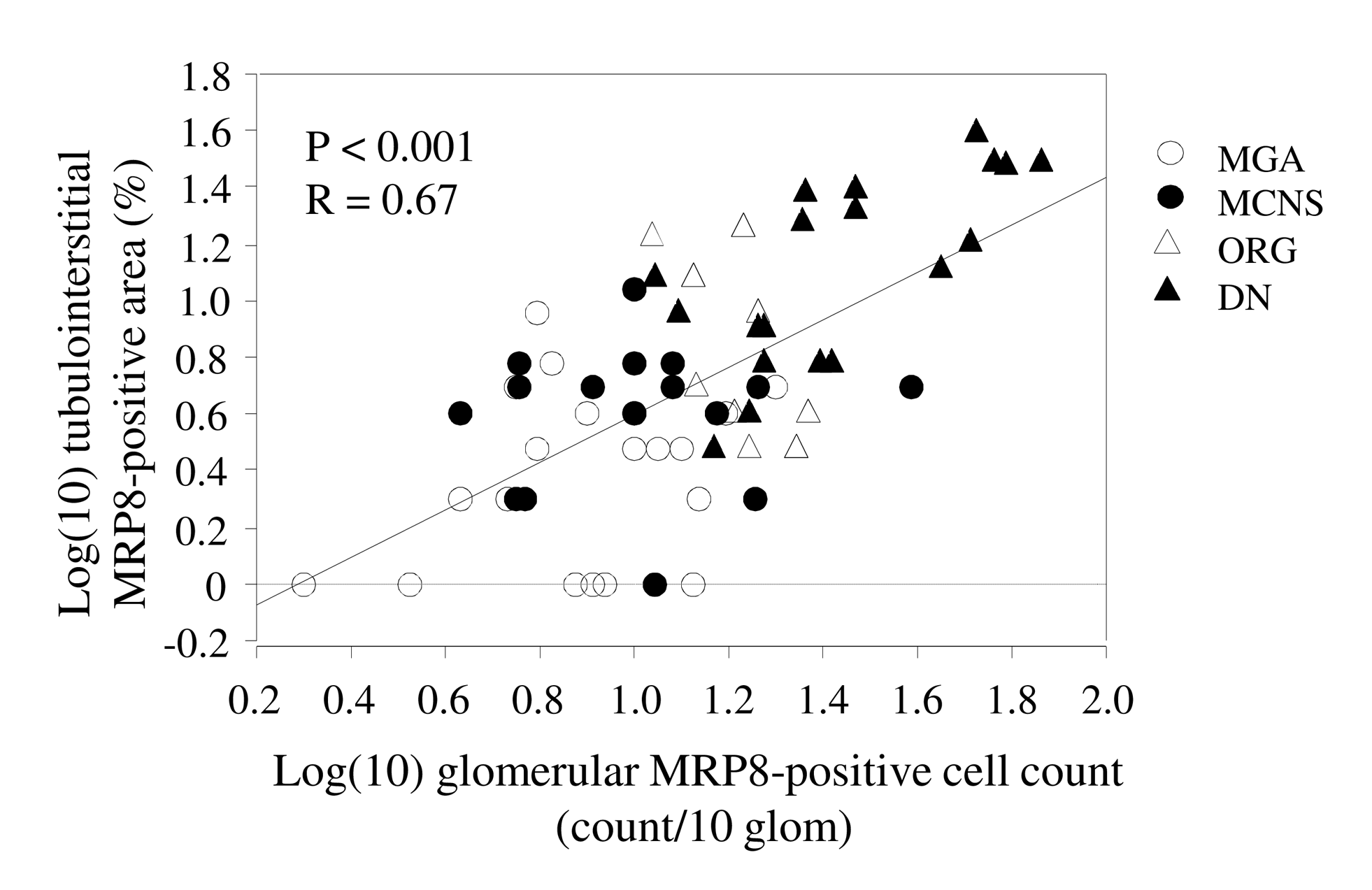

Supplement: Figure S7 — Correlation between glomerular and tubulointerstitial MRP8 expression. The log-transformed values of MRP8 signals were used. (TIF) [file pone.0088942.s007.tif]

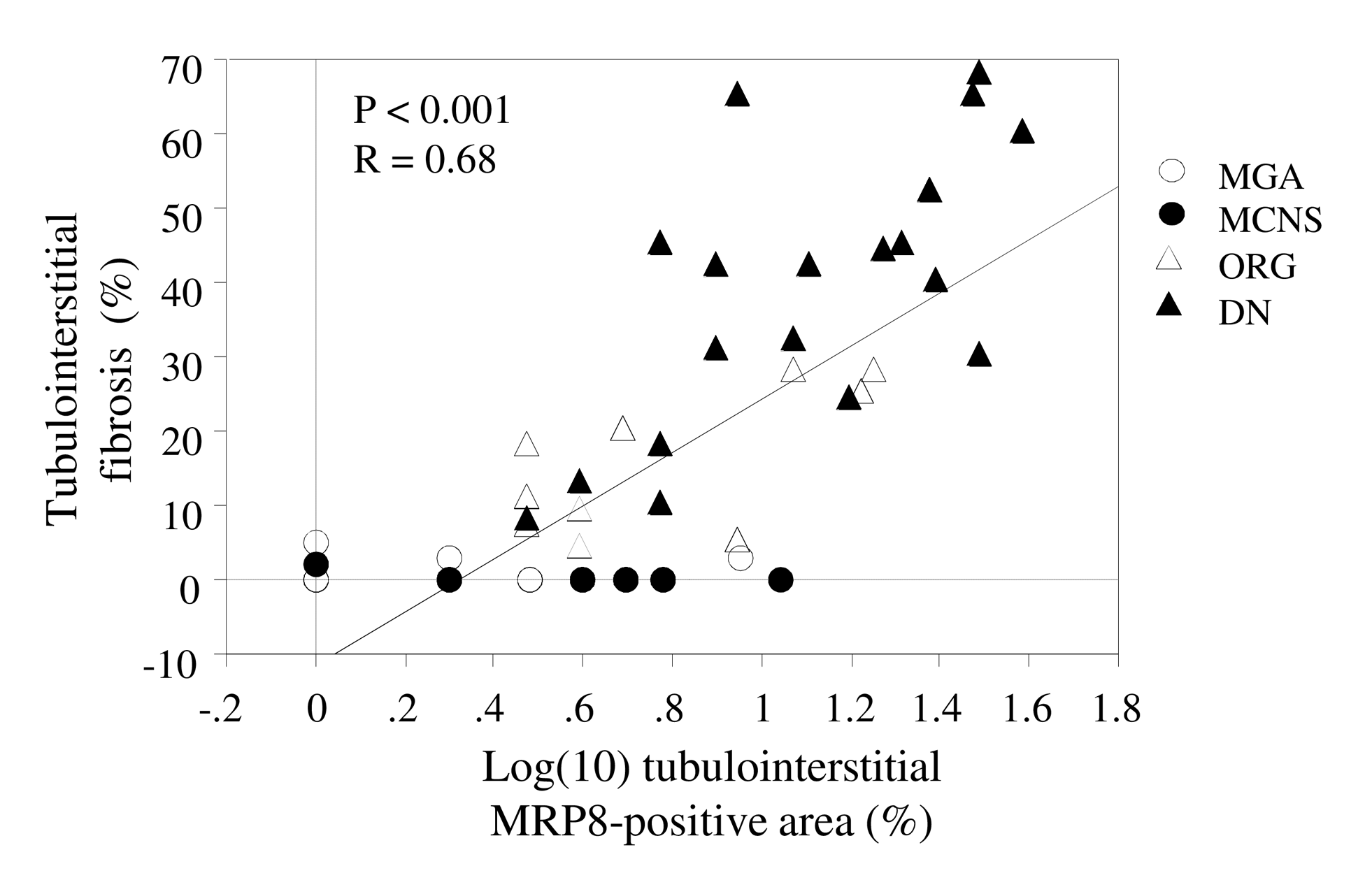

Supplement: Figure S8 — Correlation between tubulointerstitial MRP8-positive area and tubulointerstitial fibrosis. The log-transformed values of MRP8 signals were used. (TIF) [file pone.0088942.s008.tif]

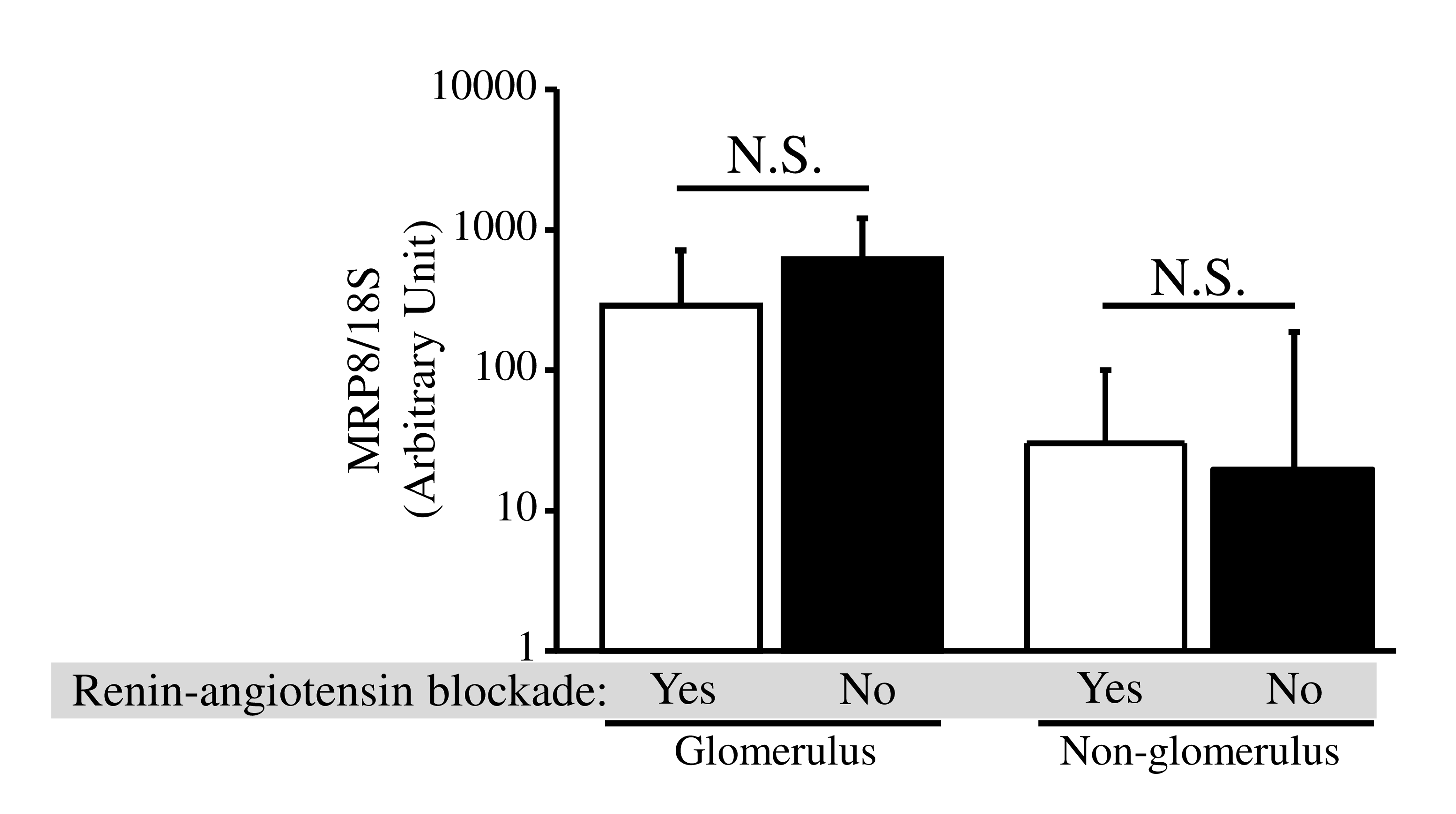

Supplement: Figure S9 — Renal mRNA expression of MRP8 in DN patients with or without renin-angiotensin blockade. N.S.: not significant. n = 15 (Yes), 6 (No). Among 22 DN cases, information about medication was not available in one patient. (TIF) [file pone.0088942.s009.tif]
