# Supplementary material for: Predictive Significance of Kidney Myeloid-Related Protein 8 Expression in Patients with Obesity- or Type 2 Diabetes-Associated Kidney Diseases
Source: PLoS One. 2014 Feb 18;9(2):e88942. doi: 10.1371/journal.pone.0088942 (PMC3928329; doi:10.1371/journal.pone.0088942)
Supplement: File S1 — Supporting Tables. Table S1, Pathological diagnoses of all cases who underwent renal biopsy at Department of Medicine and Clinical Science, Kyoto University Hospital between 2000 and 2011. Table S2, Primer and probe sequences for TaqMan real-time RT-PCR. Table S3, Logistic regression analysis for the occurrence of renal event within a year. (DOC) [file pone.0088942.s010.doc]

**Supplementary Tables**

**Table S1.** Pathological diagnoses of all cases who underwent renal biopsy at Department of Medicine and Clinical Science, Kyoto University Hospital between 2000 and 2011.

| **Pathological diagnosis** | **Number** |
| --- | --- |
| Minor glomerular abnormality | 27 |
| Minimal change nephrotic syndrome | 21 |
| Obesity-related glomerulopathy | 12 |
| Diabetic nephropathy | 23 |
| IgA nephropathy | 97 |
| Membranous nephropathy | 37 |
| Membrano-proliferative glomerulonephritis | 21 |
| Focal-segmental glomerulosclerosis | 16 |
| Crescentic glomerulonephritis | 15 |
| Lupus nephritis | 25 |
| Others | 61 |
| Total | 355 |

**Table S2.** Primer and probe sequences for TaqMan real-time RT-PCR.

| Gene | Forward primer | Reverse primer |
| --- | --- | --- |
| Human MRP8 | 5'-ACCACAAGTACTCCCTGATAAAGG-3' | 5'-CACCATCAGTGTTGATATCCAACTC-3' |
| Mouse IL-1β | 5'-TCGTGCTGTCGGACCCATA-3' | 5'-ACAGGTATTTTGTCGTTGCTTGG-3' |
| Mouse TNFα | 5'-AAGGCTGCCCCGACTACG-3' | 5'-AGGTTGACTTTCTCCTGGTATGAG-3' |
| Mouse MRP8 | 5'-ATCCTTTGTCAGCTCCGTCTTC-3' | 5'-GGGCATGGTGATTTCCTTGTATATT-3' |

| Gene | Probe |
| --- | --- |
| Human MRP8 | 5'-FAM-TTTCCATGCCGTCTACAGGGATGACCT-TAMRA-3' |
| Mouse IL-1β | 5'-FAM-AGCTGAAAGCTCTCCACCTCAATGGACA-TAMRA-3' |
| Mouse TNFα | 5'-FAM-AGGTTGACTTTCTCCTGGTATGAG-TAMRA-3' |
| Mouse MRP8 | 5'-FAM-ATCTTTCGTGACAATGCCGTCTGAACTGGA-TAMRA-3' |

**Table S3.** Logistic regression analysis for the occurrence of renal event within a year.

|  | Renal Event | | | | | | | | | |
| --- | --- | --- | --- | --- | --- | --- | --- | --- | --- | --- |
|  | Univariate | | | Multivariate | | | | | | |
|  |  | | | Model 1 | | | | Model 2 | | |
|  | OR (95%CI) | | P | | OR (95%CI) | P | | OR (95% CI) | | P |
| Sex (male) | 2.25 (0.40-12.6) | 0.36 | |  | |  | |  | |  |
| Age (years) | 1.04 (0.99-1.09) | 0.17 | |  | |  | |  | |  |
| Diabetes duration (years) | 1.01 (0.90-1.14) | 0.84 | |  | |  | |  | |  |
| BMI (kg/ m^2^) | 1.09 (0.96-1.25) | 0.19 | |  | |  | |  | |  |
| HbA1c (NGSP, %) | 0.78 (0.37-1.74) | 0.57 | |  | |  | |  | |  |
| Systolic BP (mmHg) | 1.08 (1.03-1.13) | 0.003 | | 0.88 (0.73-1.08) | | | 0.22 | | 0.94 (0.79-1.12) | 0.48 |
| Diastolic BP (mmHg) | 1.10 (1.02-1.19) | 0.01 | |  | |  | |  | |  |
| Urinary protein (g/gCr) | 1.29 (1.06-1.57) | 0.01 | | 1.03 (0.57-1.86) | | 0.91 | | 1.23 (0.74-2.04) | | 0.41 |
| Creatinine (mg/dl) | 21.0 (3.47-126.6) | 0.001 | | 11.4 (0.10-1382.6) | | 0.32 | | 1.79 (0.02-181.1) | | 0.80 |
| eGFR (ml/min/1.73m^2^) | 0.92 (0.87-0.97) | 0.004 | |  | |  | |  | |  |
| BUN (mg/dl) | 1.27 (1.09-1.48) | 0.002 | |  | |  | |  | |  |
| Total protein (g/dl) | 0.74 (0.39-1.40) | 0.36 | |  | |  | |  | |  |
| Albumin (g/dl) | 0.65 (0.31-1.35) | 0.25 | |  | |  | |  | |  |
| Total cholesterol (mg/dl) | 1.00 (0.99-1.01) | 0.72 | |  | |  | |  | |  |
| Triglyceride (mg/dl) | 1.00 (0.99-1.01) | 0.33 | |  | |  | |  | |  |
| HDL cholesterol (mg/dl) | 0.94 (0.88-1.00) | 0.57 | |  | |  | |  | |  |
| LDL cholesterol (mg/dl) | 1.00 (0.99-1.01) | 0.99 | |  | |  | |  | |  |
| CRP (mg/dl) | 0.79 (0.17-3.81) | 0.77 | |  | |  | |  | |  |
| Global GS (%) | 1.08 (1.03-1.13) | 0.002 | | 1.04 (0.92-1.19) | | 0.50 | | 1.03 (0.91-1.18) | | 0.61 |
| TI fibrosis (%) | 1.10 (1.04-1.17) | 0.001 | | 1.09 (0.90-1.31) | | 0.38 | | 1.07 (0.84-1.36) | | 0.57 |
| Glom MRP8(+) cell count | 3.48 (1.71-7.07) | < 0.001 | | 3.13 (0.93-10.6) | | 0.12 | |  | |  |
| TI MRP8(+) area (%) | 1.33 (1.12-1.59) | 0.002 | |  | |  | | 1.22 (0.95-1.57) | | 0.11 |

Odds ratio (OR) corresponds to 1 unit increase in parameters. BP: blood pressure, gCr: g creatinine, GS: glomerulosclerosis, TI: tubulointerstitial, Glom: glomerular. Unit for Glom MRP8(+) cell count is per glomelular section.
